# Clinical profile of Amnestic Early‐Onset Alzheimer’s disease in Peru: Case Series from a Neurological Care Center

**DOI:** 10.1002/alz.092996

**Published:** 2025-01-03

**Authors:** Sheila Castro‐Suarez, Erik Alberto Guevara‐Silva, Kelvin Alvarez‐Toledo, Cesar Caparo‐Zamalloa, Jonathan Adrian Zegarra‐Valdivia, Maria Meza‐Vega

**Affiliations:** ^1^ CBI en Demencias y Enfermedades Desmielinizantes del Sistema Nervioso, Instituto Nacional de Ciencias Neurológicas, Lima Peru; ^2^ Global Brain Health Institute (GBHI), University of California San Francisco, San Francisco, CA USA; ^3^ Universidad Señor de Sipán, Chiclayo Peru; ^4^ School of Medicine, Universidad Nacional Mayor de San Marcos, Lima Peru

## Abstract

**Background:**

Early‐onset Alzheimer’s disease (EOAD) is a rare and devasting form of Alzheimer disease that represents 5‐10% of the total number of Alzheimer disease (AD). It is significantly less well studied than the late‐onset form of AD. The clinical presentation is heterogeneous, the amnestic variant is the most frequent (75%). Clinical aspects of EOAD in Admixed Latin American population are lacking in the literature. We aim to describe clinical features of amnestic EOAD patients followed‐up at a Neurological healthcare center in Peru.

**Method:**

We reviewed medical records of patients with diagnosis of probable EOAD during January‐December 2023. All patients were evaluated by trained neurologists from a dementia outpatient clinic at a specialized neurologic center in Lima, Peru. Relevant information about neurocognitive, neuropsychiatric, and functional assessments were extracted from medical records with further descriptive analysis. IRB approval from local institution was obtained for this study.

**Result:**

A total of 45 patients (73% female, age at onset = 56 [50‐61] years) met the selection criteria. The 71% of the cases have more than 6 years of education. The delay for diagnosis in this group was between 2 and 5 years. A first‐degree relative with dementia was present in 15.5% of patients. Clinical and cognitive assessment profile included: MMSE average score was 13.2±6.8. The clinical manifestation characterized by anterograde episodic memory impairment were accompanied by executive disfunction (97.8%), attention and calculation impairment (84.4%), and visuospatial impairment (71%); impaired repetition of phrases and anomia were present in 67 and 44% respectively. Based on NPI the most frequent neuropsychiatric symptoms were depression (57%) irritability (51%), and anxiety (38%). Severity of dementia was CDR = 3 for 31.1%, CDR = 2 for 35.6%, CDR = 1 for 33.3%.

**Conclusion:**

The clinical features of our Peruvian amnestic EOAD cohort are mostly consistent with previous reports. There is a significant delay of EOAD diagnosis. Implementing training strategies for clinicians, neurologists, and no‐neurologists, will improve a timely and accurate diagnosis of EOAD.

